# Acetyl-DL-leucine in combination with memantine improves acquired pendular nystagmus caused by multiple sclerosis: a case report

**DOI:** 10.1007/s00415-023-11730-1

**Published:** 2023-04-27

**Authors:** O. Kremmyda, K. Feil, S. Bardins, M. Strupp

**Affiliations:** 1 Department of Neurology and German Center for Vertigo and Balance Disorders, Ludwig Maximilians University, Munich, Germany; 2 Department of Neurology and Stroke, University Hospital Tübingen, Tübingen, Germany; 3 Department of Neurology, Helios Klinikum München West, Steinerweg 5, 81241 Munich, Germany

**Keywords:** Acquired pendular nystagmus, Multiple sclerosis, Acetyl-DL-leucine, Memantine, Oscillopsia

Dear Sirs,

Acquired pendular nystagmus (APN) is a rare but serious form of spontaneous nystagmus that leads to considerable oscillopsia with reduced visual acuity, postural imbalance, and impairment of functioning and quality of life (for Ref. see [5]). The two most common causes are pontine hemorrhage and multiple sclerosis (MS). In contrast to, for instance, upbeat nystagmus, APN does not spontaneously improve over time and patients require treatment. Although the disease has a high burden and its pathophysiology is known, there is so far no established treatment for APN. In some patients, memantine or gabapentin [7, 8] caused a benefit, independent of APN aetiology, although, in our clinical experience, therapy efforts can often be frustrating.

Acetyl-DL-leucine (ADLL, Tanganil™) is a modified amino acid that has been an emerging therapeutic option for various neurological disorders in recent years. One mechanism of action is that it normalizes membrane potential and neuronal excitability [10] and it improves the metabolic state of cells [1, 3, 4]. Here we present its effects in a patient with debilitating APN due to MS who responded to a combined treatment with memantine and ADLL.

A 37-year-old female patient had been suffering from secondary progressive MS (EDSS: 6) since 2003 and from APN since 2012. For the APN, the patient had already been treated with memantine (final dosage 20 mg/d) for the past two years, but still complained of deterioration of her vision over the years. She was taking no other medication on a daily basis. Then she additionally received, as an “individual case of off-label use” (which she agreed to), 3 g per day of ADLL for three weeks, and subsequently 5 g per day. Nystagmus was measured by video-oculography (Eyeseecam®) at 0° center, ± 20° horizontal and ± 15° vertical at 10 s fixation intervals. The eye position was cleaned of eye blinks and artifacts. The areas with high acceleration (> 800 °/s^2^) and velocity (> 150 °/s) were previously removed. The velocity was prefiltered with a 30 Hz Gaussian filter. The slow phase velocity (SPV) was calculated from median filtered (with window size ¼ of the frame rate) absolute eye velocity.

After three weeks of treatment with the combination of memantine and ADLL, the patient reported an improvement of oscillopsia and stance and gait (**VIDEO 1**). No side effects were reported. Then she was asked to stop taking memantine and ADLL, which she tolerated for only two days due to a significant increase in her oscillopsia and dizziness (**VIDEO 2**). The results of the three treatment periods are summarized in Fig. 1. Memantine alone decreased horizontal nystagmus velocity from 27.3 °/s to 5 °/s, vertical nystagmus velocity from 13 °/s to 4.2 °/s, horizontal nystagmus amplitude from 1.5 ° to 0.5 ° and vertical nystagmus ampitude from 0.9 ° to 0.3 ° **(**Fig. 2).

**Fig. 1:**
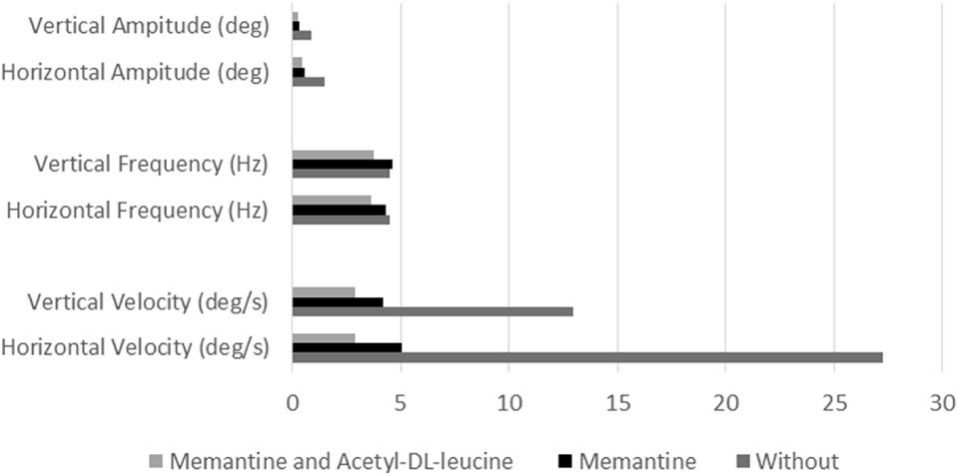
Amplitude, frequency and velocity of the acquired pendular nystagmus without medication, with memantine monotherapy, and with combined treatment

**Fig. 2:**
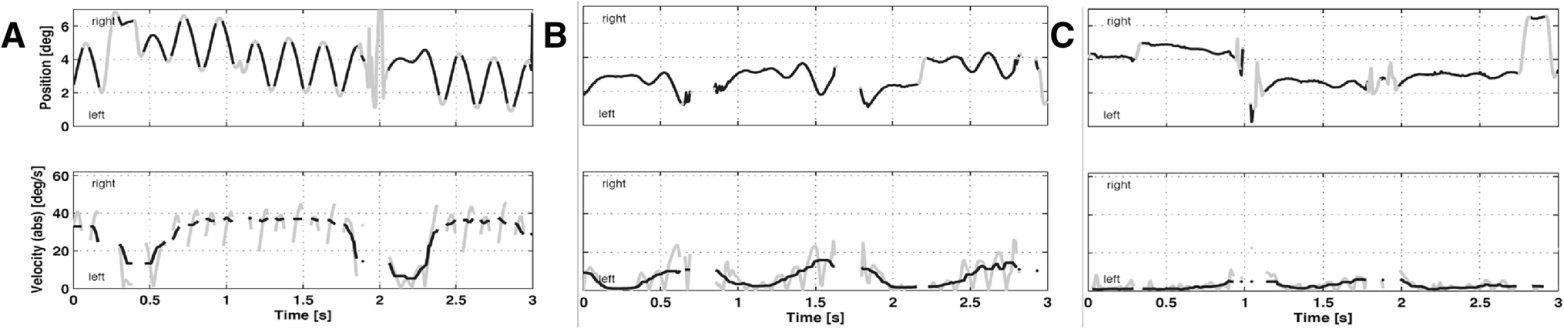
VOG recordings (horizontal traces und velocity) of the patient while fixating a central target without medication (**A**), with memantine (**B**), and with both acetyl-DL-leucine and memantine (**C**). Position: blackline: desaccaded eye position, gray line: saccades and by threshold excluded data. Velocity: black line: median filtered absolute eye velocity excluding saccades and cleared by thresholds

During the combined treatment with memantine and ADLL, nystagmus velocity was further reduced to 2.9 °/s (horizontal and vertical) (Fig. 2). This combination therapy also reduced nystagmus frequency to under 4 Hz, which memantine alone did not. The patient has now been on combined therapy with memantine (20 mg/d) and ADLL (5 g/d) for five years and is in a stable condition without reporting any side effects; symptoms worsened whenever she reduced the dosage.

Here we report on a patient with APN due to MS that significantly improved due to a combined treatment with memantine plus ADLL. In pontine hemorrhage, APN is thought to be caused by a disruption in the dentato-rubro-olivary pathway (Guillain-Mollaret-Triangle), leading to abnormal eye oscillations and oculopalatal tremor [6]. In MS, it is thought to be caused by lesions of the paramedian tract due to demyelination, which lead to instability of the neural integrator for gaze stability (in MS, it does not usually cause pseudohypertrophy of the inferior olive-like stroke related APN) [9]. Currently, memantine and gabapentin are standardly used for APN treatment in MS [8, 9]. Memantine, an NMDA receptor antagonist, evidently affects APN through the cerebellum, by reducing dentate nucleus-related oscillations [6]. ADLL may have an additional (over-additive) effect by stabilizing neuronal membrane potential and excitability, as was demonstrated by in-vitro recordings in an animal model of acute unilateral vestibulopathy. This can improve the conduction of demyelinated axons or it has a direct effect on cerebellar nuclei; the latter was demonstrated by FDG-PET studies with the L-form as the active component [2]. All in all, the combination of these two drugs with different mechanisms of action led to a significant improvement in symptoms in our patient.

We conclude that ADLL may provide a therapeutic alternative for APN therapy in MS patients and should be tested individually in these patients in combination with standard existing therapies, such as memantine since ADLL’s side effects are negligible and therapy options are so far limited.

## Supplementary Information

Below is the link to the electronic supplementary material.

Supplementary file1 (MP4 148173 KB)

Supplementary file2 (MP4 108209 KB)
